# The Values of Systemic Immune-Inflammation Index and Neutrophil–Lymphocyte Ratio in the Localized Prostate Cancer and Benign Prostate Hyperplasia: A Retrospective Clinical Study

**DOI:** 10.3389/fonc.2021.812319

**Published:** 2022-01-03

**Authors:** Shuo Wang, Yongpeng Ji, Yanyun Chen, Peng Du, Yudong Cao, Xiao Yang, Jinchao Ma, Ziyi Yu, Yong Yang

**Affiliations:** ^1^ Key Laboratory of Carcinogenesis and Translational Research (Ministry of Education), Urological Department, Peking University Cancer Hospital and Institute, Beijing, China; ^2^ Key Laboratory of Carcinogenesis and Translational Research (Ministry of Education), Anesthesiology Department, Peking University Cancer Hospital and Institute, Beijing, China

**Keywords:** prostate cancer, systemic immune-inflammation index, neutrophil–lymphocyte ratio, inflammatory markers, neutrophil ratio

## Abstract

**Purpose:**

To evaluate the diagnostic values of systemic immune-inflammation index (SII) and neutrophil–lymphocyte ratio (NLR) in patients with localized prostate cancer (PCa).

**Methods:**

Between January 2014 and December 2019, 117 patients with benign prostate hyperplasia (BPH) and 278 patients with localized PCa who underwent radical prostatectomy (RP) were included in this study. The inflammatory markers including SII, NLR, platelet–lymphocyte ratio (PLR), lymphocyte–monocyte ratio (LMR), lymphocyte ratio (LR), neutrophil ratio (NR), mean platelet volume (MPV), and red cell distribution (RDW) of these two groups were examined and analyzed. ROC curve analysis was performed to assess the discriminative ability of inflammatory markers and their combination with tPSA for PCa. The binary logistic regression model was used to evaluate the association between significant inflammatory markers and risk of PCa.

**Results:**

The pathological results from RP specimen comprised 72 (25.90%) patients with pT1, 168 (60.43%) patients with pT2, and 38 (13.67%) patients with pT3. According to Student’s *t* test, patients with PCa had higher NLR (*p* = 0.034), SII (*p* = 0.008), and NR (*p* = 0.004), and lower LR (*p* = 0.025), MPV (*p* = 0.003), and TPV (*p* = 0.022) compared with patients with BPH; the distribution of age, PLR, LMR, RDW, f/t PSA ratio, and BMI did not show any significant differences. The AUC for NLR, SII, NR, and tPSA was 0.697 (*p* = 0.015), 0.719 (*p* < 0.001), 0.647 (*p* = 0.009), and 0.708 (*p* < 0.001), with threshold values of 1.6, 471.86, 65.15%, and 12.89 ng/ml, respectively. Patients were divided into two groups according to the threshold values, respectively. By using the multivariable logistic regression models, NLR ≥ 1.6 (OR, 2.731; 95% CI, 0.937–7.961, *p* = 0.042), SII ≥ 471.86 (OR, 1.274; 95% CI 0.473–3.433; *p* = 0.033), and PSA ≥ 12.89 ng/ml (OR, 1.443; 95% CI, 0.628–3.944; *p* = 0.014) were independent risk factors associated with PCa. The AUC for combination of NLR, SII, and NR with tPSA was 0.705 (*p* < 0.001), 0.725 (*p* < 0.001), and 0.704 (*p* < 0.001), respectively.

**Conclusion:**

This study demonstrated that SII, NLR, and NR were all independent risk factors of PCa. These factors alone could provide better screen methods for PCa before biopsy. In addition, SII is a more powerful tool among these three inflammatory markers associated with PCa. Besides, combination of SII and NLR with tPSA had not much advantage compared with themselves alone.

## Introduction

Prostate-specific antigen (PSA) is the most widely used method for predicting prostate cancer (PCa); it increases the number of tumors diagnosed at early stage (1). However, it also increases the rate of overdiagnosis and overtreatment in a considerable number of patients. Besides PSA, the current available prebiopsy tools including digital rectal examination and transrectal prostate ultrasound examination are all not accurate enough to guide biopsy. To improve the accuracy of discriminating PCa, other meaningful methods are needed. In recent decades, several biomarkers of hematological indices representative of systemic immune-inflammatory responses have been verified as risk factors associated with several kinds of solid tumor (2, 3). More recently, inflammation factors including neutrophil–lymphocyte ratio (NLR), platelet–lymphocyte ratio (PLR), and neutrophil ratio (NR) have been proposed as predictors of PCa (4). NLR is an independent factor for overall survival after RP (5), and higher NLR was associated with tumor aggressiveness and higher Gleason Score (GS) in metastatic PCa (6). In addition, another novel inflammation marker, systemic immune-inflammation index (SII), has been shown to have a more powerful diagnostic and prognostic value in various kinds of tumors (7). In terms of PCa, SII was firstly reported in 2016 and considered as a prognostic factor of metastatic PCa (8), and it was an independent factor associated with the therapy efficiency of docetaxel in metastatic castration-resistant prostate cancer (mCRPC) (9). Nevertheless, to our knowledge, there have been no study reporting the diagnostic value of SII in localized PCa. Both NLR and SII are all easily measurable and inexpensive parameters that can be calculated easily from complete blood counts (CBCs).

In this study, we aimed to investigate the diagnostic value of inflammation markers including SII and NLR in localized PCa and evaluate whether they have diagnostic differences with tPSA.

## Materials and Methods

### Patients

This is a diagnostic study with a retrospective design. A total of 117 patients with BPH and 278 patients with localized PCa who underwent radical prostatectomy (RP), namely, 274 with laparoscopic RP and 4 with open RP, at Peking University Cancer Hospital and Institute between January 2014 and December 2019 were reviewed. Data related to PCa risk factors were collected, and the association between predictive factors and risk of PCa was analyzed. The factors included were age, serum tPSA value, f/t PSA ratio, total prostate volume (TPV), body mass index (BMI), and CBC-based parameters. A single pre-biopsy CBC with differential was performed as part of the routine assessment testing before biopsy simultaneously with tPSA value and its f/t ratio, and the CBC-based parameters including NLR, PLR, lymphocyte–monocyte ratio (LMR), SII, lymphocyte ratio (LR), NR, mean platelet volume (MPV), and red cell distribution (RDW) were used in this study.

### Procedure

Results of serum tPSA value, f/t PSA ratio, and CBC-based parameters were collected from just before biopsy. Ultrasound guided 13-core trans-rectal prostate biopsy technique was performed in patients with PSA > 4 ng/ml in our institute. All specimens were assessed by a sophisticated pathologist at our institute. The patients were pathologically classified into BPH and PCa groups. MRI, emission computed tomography (ECT), or CT was performed before surgery to confirm no bone, distant organ, or lymph node metastasis in patients with PCa. Laparoscopic RP or open RP was performed in patients with PCa at least 30 days after biopsy. Extra-fascial radical prostatectomy through an extraperitoneal approach was performed by skilled and experienced surgeons in our institute according to the technique of Walsh et al. (10).

### Variables

Pathologic GS were recorded and prostate was measured in 3-dimensional aspects, and its volume was estimated with the modified ellipsoid formulation in cm^3^ (0.523 [length × width × height]) after surgery; patients were staged according to the 2010 American Joint Committee on Cancer system (AJCC, pathologic stage T1–T4) (11), and tumors were classified into low-, intermediate-, and high-grade group according to D’Amico risk classification (12). CBC-based parameters including LR, NR, MPV, and RDW were evaluated with the peripheral blood samples, and NLR, PLR, LMR, and SII were calculated by using the number of blood cell counts based on systemic markers of inflammation. SII (SII = platelet × neutrophil/lymphocyte) has been presented as a combination of NLR and PLR (13, 14). Then, we statistically evaluated the association of clinical and CBC base parameters with PCa.

### Statistical Analysis

Measurement data conforming to normal distribution analyzed by Shapiro–Wilk test are presented as mean ± SD, and independent sample *t*-test and Box-plot graphics are used for comparison between groups. ROC curve analyses were performed to assess the discriminative ability of the inflammatory markers and their combination with tPSA for PCa. The cutoff points for markers were defined by a criterion based on Youden’s index defined as YI_(C)_ = max c [Se_(C)_ + SP_(C)_-1] and corresponding specificity–sensitivity levels were provided. The binary logistic regression model (univariate and multivariate analysis) was used to evaluate the association between significant factors and risk of PCa, which were all compared with reference group (Ref). The software used to run the analysis was IBM-SPSS version 20. All tests were two-sided, and *p* < 0.05 was considered to be the threshold for statistically meaningful differences.

## Results

### Patients’ Clinicopathologic Characteristics

A total of 395 patients were enrolled into the study, namely, 117 with BPH and 278 with localized PCa. The median values of clinical factors were 65.56 ± 5.93 years for age, 39.1 ± 23.56 ml for TPV, and 24.57 for BMI. Among patients with PCa, 72 (25.90%) were pT1, 168 (60.43%) were pT2, and 38 (13.67%) were pT3 according to the AJCC system; 84 (30.22%) were low risk (GS ≤ 6), 120 (43.17%) were intermediate risk (GS = 7), and 74 (26.62%) were high risk (GS ≥ 8) according to D’ Amico risk criteria (12).

### Analysis of Clinical and CBC-Based Parameters

**Figure 1:**
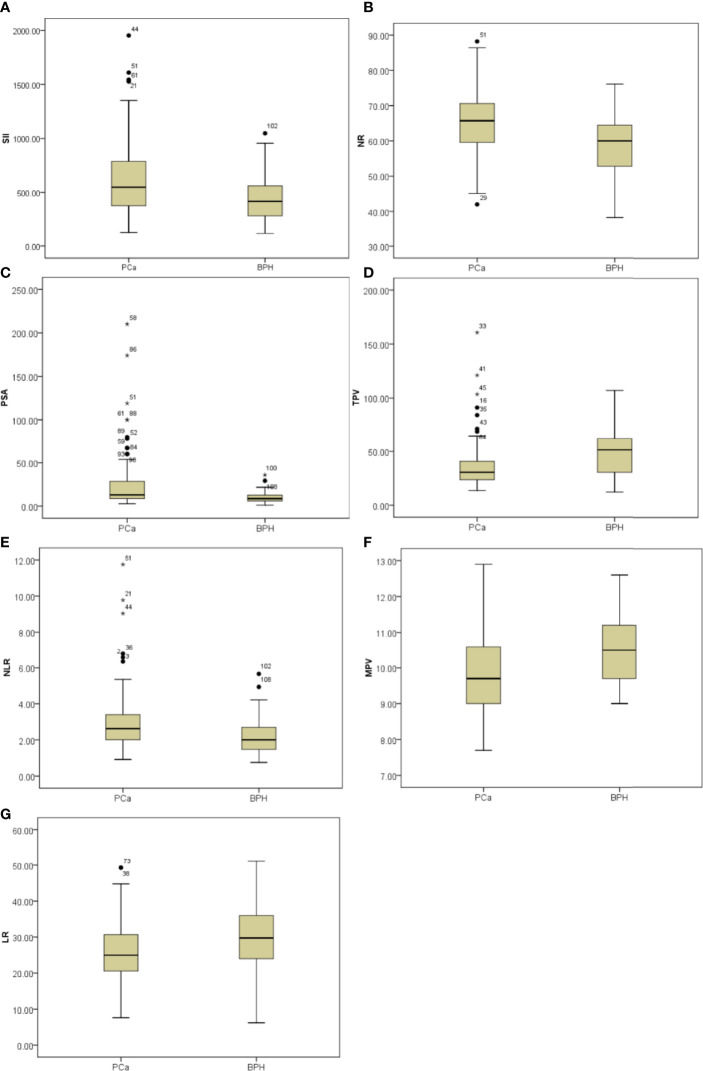
Box-blot graphics of inflammatory markers including SII, NR, PSA, TPV, NLR, MPV, and LR for patients with PCa and patients with BPH. **(A–C, E)** SII, NR, PSA, and NLR in the PCa group were significantly higher than in the BPH group, *p* = 0.008, *p* = 0.004, *p* = 0.006, *p* = 0.034, respectively. **(D, F, G)** TPV, MPV, and LR in the PCa group were significantly lower than in the BPH group, *p* = 0.022, *p* = 0.003, *p* = 0.025, respectively.

Initially, clinical and CBC-based parameters were analyzed and compared among patients with PCa and patients with BPH by Student’s *t-*test. Patients with PCa had higher NLR (*p* = 0.034), SII (*p* = 0.008), and NR (*p* = 0.004) and lower LR (*p* = 0.025), MPV (*p* = 0.003), and TPV (*p* = 0.022) compared with patients with BPH, but the distribution of age, PLR, LMR, RDW, f/t PSA ratio, and BMI did not show any significant differences as shown in **Figure 1** and **Table 1**.

**Table 1:** Comparison of clinical and CBC-based parameters with respect to groups.

Variables	PCa group (*n* = 278)	BPH group (*n* = 117)	*p*-value
Age (years)	66.1 ± 5.93	64.02 ± 8.66	0.115
NLR (%)	2.94 ± 1.74	2.28 ± 1.12	0.034
PLR (%)	145.76 ± 52.46	129.04 ± 39.08	0.083
LMR (%)	4.28 ± 1.96	4.62 ± 2.29	0.394
SII (%)	613.28 ± 346.93	448.47 ± 202.28	0.008
LR (%)	26.08 ± 8.18	29.83 ± 9.48	0.025
NR (%)	64.86 ± 9.18	59.66 ± 9.04	0.004
MPV (10^3^/μl)	9.82 ± 1.09	10.45 ± 0.96	0.003
RDW (%)	12.83 ± 0.78	12.83 ± 0.56	0.991
tPSA (ng/ml)	25.70 ± 33.16	10.15 ± 6.96	0.006
f/t ratio	0.15 ± 0.24	0.16 ± 0.08	0.858
TPV (ml)	36.62 ± 23.15	49.36 ± 22.97	0.022
BMI	24.35 ± 4.14	25.12 ± 3.05	0.360

NLR, neutrophil–lymphocyte ratio; PLR, platelet–lymphocyte ratio; LMR, lymphocyte–monocyte ratio; SII, systemic immune-inflammation index; LR, lymphocyte ratio; NR, neutrophil ratio; MPV, mean platelet volume; RDW, red cell distribution; CBC, complete blood count; PSA, prostate cancer specific antigen; TPV, total prostate volume; BPC, biopsy positive cores; BMI, body mass index.

**Figure 2:**
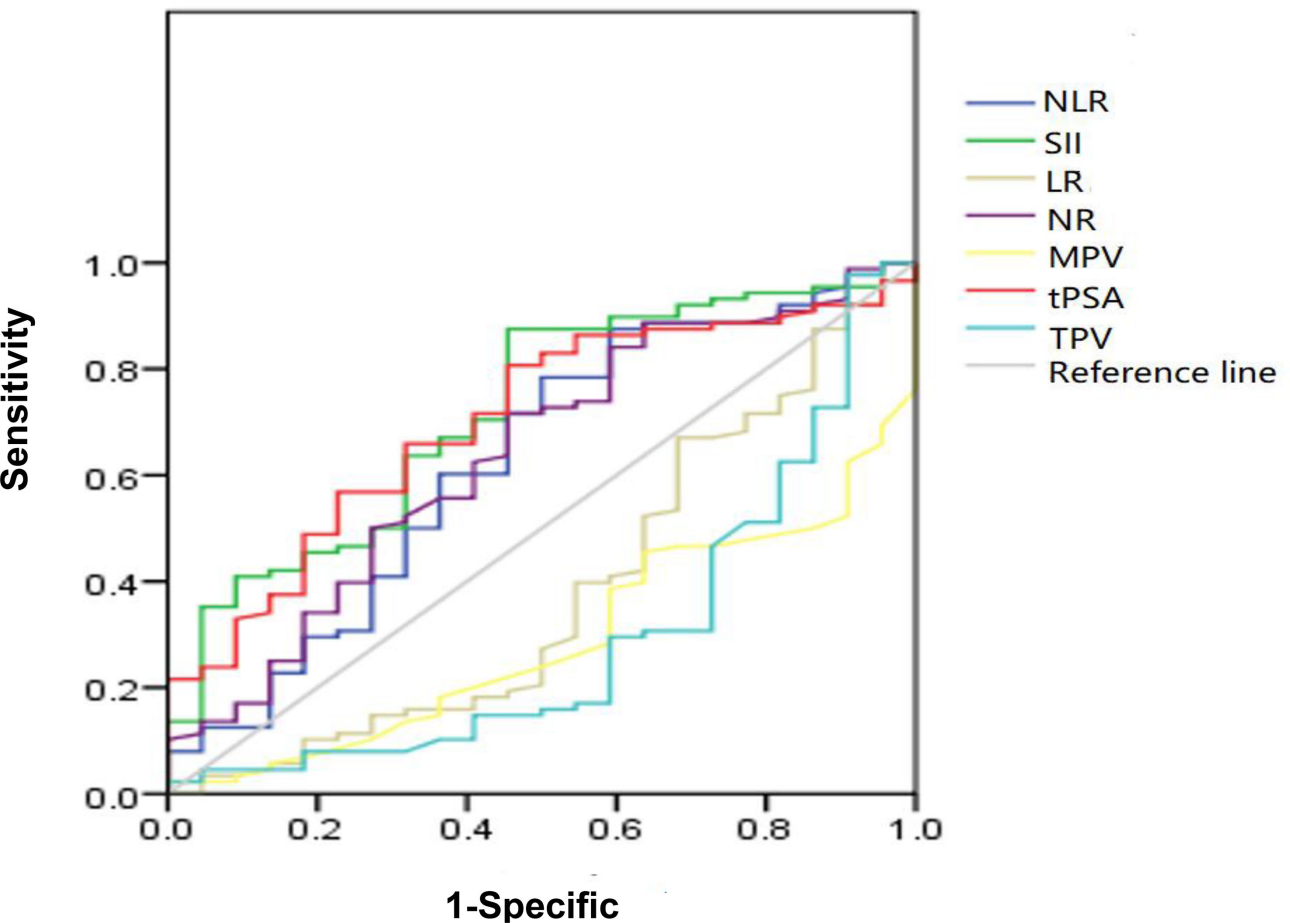
Diagnostic role of SII, NLR, NR, tPSA, TPV, MPV, and LR in detecting localized PCa by ROC curve analysis. Among inflammatory markers, SII had largest AUC of 0.719, the second was NLR with AUC of 0.697, the third was NR with AUC of 0.647; the AUC of tPSA was 0.708; both SII and NLR got the comparable AUC with tPSA.

The ROC curve for NLR, SII, NR, LR, MPV, and TPV was plotted in the diagnosis of PCa as shown in **Table 2** and **Figure 2**. In patients with PCa, AUC for NLR was 0.697, which was significantly lower than 0.05 (*p* = 0.015), with a threshold value of 1.6, a sensitivity of 87.1%, and a specificity 41.7%; AUC for SII was 0.719, which was significantly lower than 0.05 (*p* < 0.001), with a threshold value of 471.86, a sensitivity of 51.4%, and a specificity of 85.7%; AUC for NR was 0.647, which was significantly lower than 0.05 (*p* = 0.009) with a threshold value of 65.15%, a sensitivity of 49.5%, and a specificity of 77.8%; AUC for tPSA was 0.708, which was significantly lower than 0.05 (*p* < 0.001) with a threshold value of 12.89 ng/ml, a sensitivity of 51.5%, and a specificity of 83.3%; the AUC for LR, MPV and TPV were 0.375, 0.330, and 0.300, respectively, which were significantly lower than 0.05 (*p* = 0.026, *p* = 0.003, and *p* = 0.004, respectively). Together, we found a comparable value between NLR, SII, and tPSA; NLR and SII exhibited good differential diagnosis potential, which could be used as adjuvant tool in diagnosing PCa.

**Table 2:** Cutoff, AUC, sensitivity, and specificity values of NLR, SII, LR, NR, MPV, tPSA, and TPV.

Variables	AUC	Cutoff	Sensitivity	Specificity	95% CI	*p*-value
NLR	0.697	≥1.60	87.1%	41.7%	0.528–0.746	0.015
SII	0.719	≥471.86	51.4%	85.7%	0.627–0.817	<0.001
LR	0.375	≥15.12%	93.1%	8.3%	0.264–0.486	0.026
NR	0.647	≥65.15%	49.5%	77.8%	0.542–0.753	0.009
MPV	0.330	<12.7	20%	100%	0.235–0.425	0.003
tPSA	0.708	≥12.89	51.5%	83.3%	0.616–0.801	<0.001
TPV	0.300	<15.02	97.8%	9.1%	0.168–0.432	0.004

AUC, Area under the curve; NLR, neutrophil–lymphocyte ratio; SII, systemic immune-inflammation index; TPV, total prostate volume; MPV, mean platelet volume; NR, neutrophil ratio; LR, lymphocyte ratio.

Then, patients were divided into 2 groups according to the threshold value of NLR, SII, NR, and tPSA; univariable and multivariable logistic regression models were used to evaluate the association between factors and risk of PCa. In univariable analysis, NLR ≥ 1.6 (OR, 7.818; 95% CI, 4.174–14.644, *p* < 0.001), SII ≥ 471.86 (OR, 1.694; 95% CI, 1.122–2.558, *p* = 0.003), NR ≥ 65.15% (OR, 1.424; CI, 0.913–2.223, *p* < 0.001), and PSA ≥ 12.89 ng/ml (OR, 1.516; 95% CI, 0.963–2.386, *p* < 0.001) were risk factors associated with PCa as shown in **Table 3**. In multivariable analysis, NLR ≥ 1.6 (OR, 2.731; 95% CI, 0.937–7.961, *p* = 0.042), SII ≥ 471.86 (OR, 1.274; 95% CI 0.473–3.433; *p* = 0.033), and PSA ≥ 12.89 ng/ml (OR, 1.443; 95% CI, 0.628–3.944; *p* = 0.014) were all independent risk factors associated with PCa as shown in **Table 3**.

**Table 3:** Univariable and multivariable analyses for predicting localized prostate cancer.

	Univariable analysis PCa vs. BPH			Multivariable analysis PCa vs. BPH		
	OR	95% CI	*p*-value	OR	95% CI	*p*-value
NLR						
<1.6	1 (Ref)	1 (Ref)		1 (Ref)	1 (Ref)	
≥1.6	7.818	4.174–14.644	<0.001	2.731	0.937–7.961	0.042
SII						
<471.86	1 (Ref)	1 (Ref)		1 (Ref)	1 (Ref)	
≥471.86	1.694	1.122–2.558	0.003	1.274	0.473–3.433	0.033
NR						
<65.15%	1 (Ref)	1 (Ref)		1 (Ref)	1 (Ref)	
≥65.15%	1.424	0.913–2.223	<0.001	2.676	0.93–7.696	0.051
tPSA (ng/ml)						
<12.89	1 (Ref)	1 (Ref)		1 (Ref)	1 (Ref)	
≥12.89	1.516	0.963–2.386	<0.001	1.443	0.628–3.944	0.014

Ref, reference; NLR, neutrophil–lymphocyte ratio; SII, systemic immune-inflammation index; NR, neutrophil ratio; PSA, prostate-specific antigen.

### ROC Curve Analysis of SII, NLR, and NR Combined With tPSA

**Figure 3:**
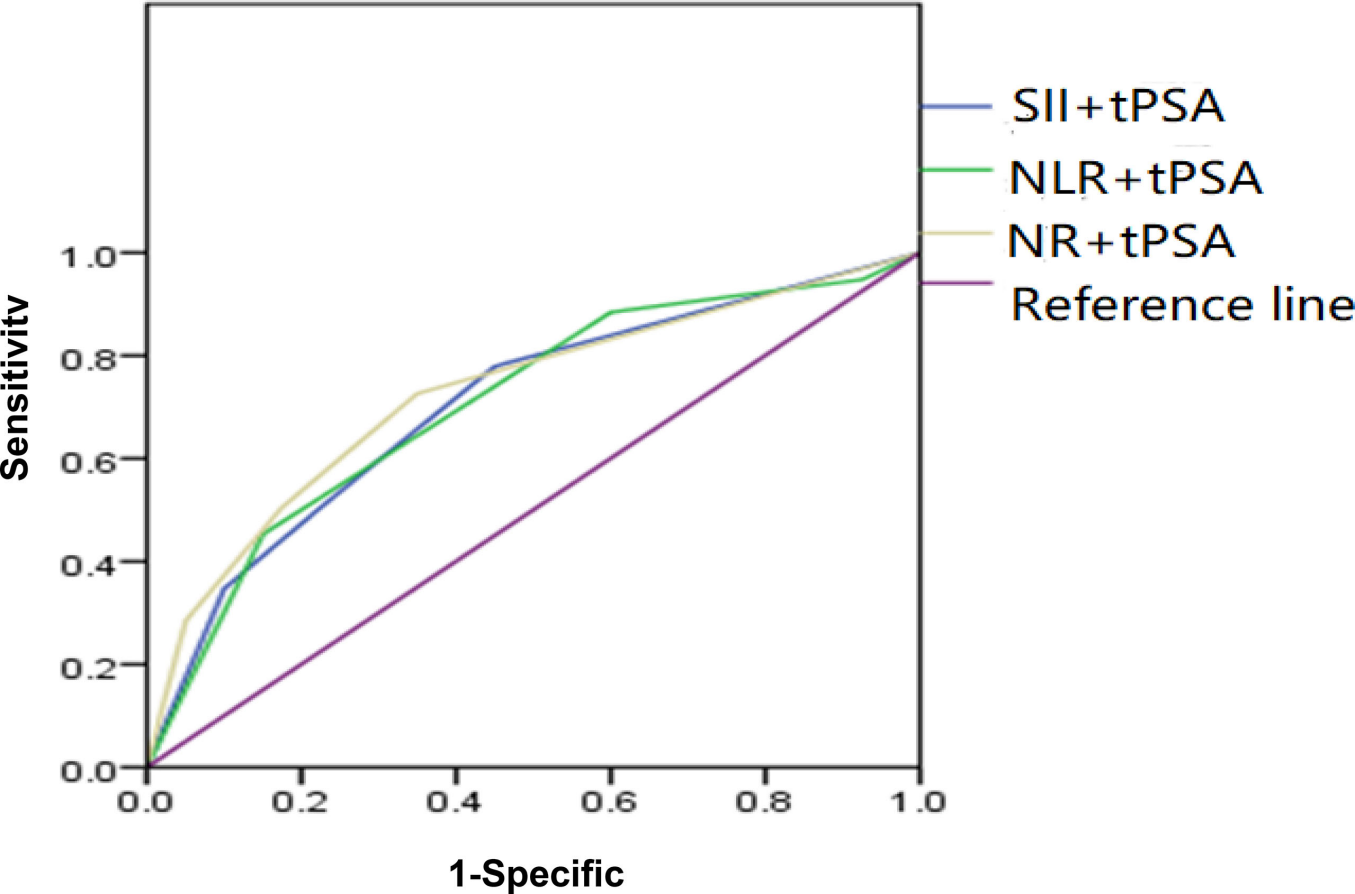
ROC analysis of SII, NLR, and NR combined with tPSA. SII + tPSA got the largest AUC of 0.725, the second was NLR + tPSA with an AUC of 0.705, and the third was NR + tPSA with an AUC of 0.704.

Depending on the threshold values of SII, NLR, NR, and tPSA, we classified patients and assessed clinical usefulness of tPSA + SII, tPSA + NLR, and tPSA + NR. The results revealed that AUC for tPSA + SII was 0.725, which was significantly lower than 0.05 (*p* < 0.001) with a sensitivity of 55.5% and a specificity of 82.5%; AUC for tPSA + NLR was 0.705, which was significantly lower than 0.05 (*p* < 0.001) with a sensitivity of 52.3% and a specificity of 85%; AUC for tPSA + NR was 0.704, which was significantly lower than 0.05 (*p* < 0.001) with a sensitivity of 50.5% and a specificity of 77.5% as shown in **Table 4** and **Figure 3**.

**Table 4:** AUC, sensitivity, and specificity values of SII, NLR, and NR combined with tPSA.

Variables	AUC	Sensitivity	Specificity	95% CI	*p*-value
SII + tPSA	0.725	55.5%	82.5%	0.635–0.815	<0.001
NLR + tPSA	0.705	52.3%	85%	0.610–0.801	<0.001
NR + tPSA	0.704	50.5%	77.5%	0.610–0.799	<0.001

PSA, prostate-specific antigen; NLR, neutrophil–lymphocyte ratio; SII, systemic immune-inflammation index; NR, neutrophil ratio.

## Discussion

This study evaluated the diagnostic value of inflammatory factors and their potential in selecting patients for prostate biopsy. Currently, the most common preoperative tools used for predicting the occurrence of PCa is PSA. The men were suspected with PCa when PSA is above 4 ng/ml (15). However, there are some limitations for the use of PSA to predict PCa, and it is demonstrated that up to 20% of patients with PCa may be misdiagnosed at their first biopsy guiding by PSA (16). In order to increase the accuracy rate of biopsy, more applicable and available ways are needed. In recent years, there is increasing evidence correlating the presence of systemic inflammation with occurrence of several solid tumors (17–19). Inflammation might influence the pathogenesis of cancers by several different methods (20). Inflammatory response in microenvironment might play a key role in the occurrence of tumors (21). On this basis, systemic inflammatory markers might be potentially used as a prognostic factor in cancer patients (22). As most usually used systemic inflammatory markers, NLR, PLR, and NR have drawn much attention owing to their advantage of convenience and low cost. Especially for NLR, it could serve as a biomarker to predict the true nature of the mass; the elevated NLR also indicated poor outcomes of malignancy in several kinds of tumors (23). However, recently, some studies believed that SII is a more powerful tool associated with the occurrence or progression of tumors compared with NLR or NR (9, 24). As a novel inflammatory index, SII was first described by Hu et al. in 2014 (13). High SII means thrombocythemia, lymphopenia, or neutrophilia, suggesting an elevated non-specific inflammatory status and weak adaptive immune response in patients, which will cause tumors escaping from immune surveillance, and it was considered a powerful prognostic index in various kinds of tumors including hepatocarcinoma (13), renal cell cancer (8), and gastric cancer (25).

In terms of PCa, several studies demonstrated an association of inflammatory factors with the relative risk and disease aggressiveness of PCa (26). As a cancer-related-systemic inflammatory marker, NLR may predict PCa with or without combination with f/t PSA ratio (6). Jang WS showed that preoperative NLR is an independent prognostic factor for cancer-specific survival after RP (5). Other studies also demonstrated that higher NLR was associated with higher Gleason Score (6) and could be the risk factor for biochemical recurrence after RP (27). Instead of NLR, the baseline neutrophil cell and lymphocyte cell were reported to be better choices for predicting progression of PCa (28). All the above studies indicated that NLR might be a useful biomarker associated with risk of PCa. In our study, NLR was demonstrated to be a reliable independent factor associated with localized PCa, which was consistent with results of previous studies, but its diagnostic value might be inferior to SII or PSA due to its lower AUC area. Then, we combined NLR with tPSA to calculate its AUC area, but its diagnostic value is still inferior compared with SII or PSA alone.

To our knowledge, there is little or no information in the literature regarding the diagnostic value of SII in localized PCa. The first study reporting the association of SII with PCa was conducted in 2016, which reported that SII could be a prognostic marker in patients with metastatic PCa; the cutoff value of SII was determined to be 535.0 (8). Another study published recently demonstrated that SII could be an effective marker for predicting the prognosis of patients with mCRPC treated with docetaxel (7). In this study, for the first time in the literature, the role of SII in predicting PCa was analyzed and the results indicated that SII had diagnostic value in the detection of localized PCa; the cutoff value of SII was determined to be 471.86, which was similar to that reported by Lolli et al. (8). Using the ROC curve and logistic regression analysis, we demonstrated that the SII, NLR, and NR were all good methods used in terms of diagnostic yield. These factors were all independent factors associated with PCa, while SII seemed to be a more favorable choice because it had the largest AUC area of 0.719 compared with NLR of 0.697, NR of 0.647, and tPSA of 0.708. Although we combined SII, NLR, and tPSA to evaluate their association with PCa, they have no obvious advantages compared with SII, NLR, or tPSA alone since the AUC areas were similar between them. We concluded that SII is one of the most important inflammatory biomarkers for the detection of PCa, and guiding decisions to prostate biopsy need not be combined with tPSA.

This study still has some limitations: First, the retrospective nature of the study. Second, the usage of single preoperative blood samples is less reliable; it can be strengthened by collecting different pre-biopsy sets of blood samples from each patient within a short period. Third, the large difference of sample size in two groups may cause bias; more sample size is required in the BPH group in further studies.

## Conclusion

In conclusion, this study demonstrated that SII, NLR, and NR alone were all independent risk factors of localized PCa, as they showed superior performance in detecting PCa. These factors could provide better screen methods associated with PCa before biopsy. In addition, SII is a more powerful tool among these three inflammatory factors for the diagnosis of PCa. Besides, combination of SII and NLR with tPSA had no advantage compared with themselves alone since the AUC areas were similar. More stratification models and prospective studies are needed.

## Data Availability Statement

The raw data supporting the conclusions of this article will be made available by the authors, without undue reservation.

## Ethics Statement

The studies involving human participants were reviewed and approved by the Institutional Review Board of Peking University Cancer Hospital and Institute. Written informed consent for participation was not required for this study in accordance with the national legislation and the institutional requirements.

## Author Contributions

PD and SW designed the study. SW, YJ, and YYC made the same contribution in this study as the first co-author. SW, YJ, YYC, YDC, XY, JM, ZY, PD, and YY performed the study and analyzed the data. PD, SW, YJ, and YYC wrote the manuscript draft and revised the manuscript. All authors contributed to the article and approved the submitted version.

## Funding

Capital Clinical Characteristics and Application Research Project (Project No. Z18110700170000).

## Conflict of Interest

The authors declare that the research was conducted in the absence of any commercial or financial relationships that could be construed as a potential conflict of interest.

## Publisher’s Note

All claims expressed in this article are solely those of the authors and do not necessarily represent those of their affiliated organizations, or those of the publisher, the editors and the reviewers. Any product that may be evaluated in this article, or claim that may be made by its manufacturer, is not guaranteed or endorsed by the publisher.
